# Independent and joint effects of sleep duration and sleep quality on suboptimal self-rated health in medical students: A cross-sectional study

**DOI:** 10.3389/fpubh.2022.957409

**Published:** 2022-10-06

**Authors:** Pan Ding, Jinyong Li, Huajian Chen, Chongzhou Zhong, Xiaoli Ye, Hongying Shi

**Affiliations:** ^1^Department of Preventive Medicine, School of Public Health and Management, Wenzhou Medical University, Wenzhou, China; ^2^Renji College, Wenzhou Medical University, Wenzhou, China; ^3^Propaganda Department, Wenzhou Medical University, Wenzhou, China

**Keywords:** sleep duration, sleep quality, joint effects, suboptimal self-rated health, medical students

## Abstract

**Objective:**

Studies on the association between sleep behavior and health often ignored the confounding effects of biorhythm-related factors. This study aims to explore the independent and joint effects of sleep duration and sleep quality on suboptimal self-rated health (SRH) in medical students.

**Methods:**

Cross-sectional study. Proportional stratified cluster sampling was used to randomly recruit students from various medical specialties at a medical university in eastern China. Our questionnaire mainly included information on basic demographic characteristics, SRH, sleep behavior, and biorhythm-related factors. The independent and joint effects of sleep duration and sleep quality on suboptimal SRH were assessed by logistic regression after controlling for potential confounders.

**Results:**

Of 1,524 medical students (mean age = 19.9 years, SD = 1.2 years; 59.1% female), 652 (42.8%) had suboptimal SRH. Most medical students (51.5%) slept for 7 h/night, followed by ≥8 (29.1%) and ≤ 6 h (19.4%). After adjusting for basic demographic characteristics and biorhythm-related factors, compared with students who slept for ≥8 h/night, the adjusted *ORs* (95%*CI*) for those who slept 7 and ≤ 6 h/night were 1.36 (1.03, 1.81) and 2.28 (1.60, 3.26), respectively (*P* < 0.001 for trend); compared with those who had good sleep quality, the adjusted *ORs* (95%CI) for those who had fair and poor sleep quality were 4.12 (3.11, 5.45) and 11.60 (6.57, 20.46), respectively (*P* < 0.001 for trend). Further, compared with those who slept for ≥8 h/night and good sleep quality, those who slept ≤ 6 h and poor sleep quality had the highest odds of suboptimal SRH (*OR* 24.25, 95%*CI* 8.73, 67.34).

**Conclusions:**

Short sleep and poor sleep quality were independently and jointly associated with higher odds of suboptimal SRH among medical students.

## Introduction

Self-rated health (SRH) comprehensively assesses mental and physical health, which is an easily securable and widely used global health indicator (1). It has been shown to be an important predictor for future morbidity and even mortality (2). Previous studies have shown the suboptimal SRH rates was 33.2–38.6% in the general population (1, 3, 4), 35.6–54.6% in college students (5, 6). And the SRH status of medical students was rarely reported. The health status of medical students was often less optimistic than that of the general population due to their heavy academic burden and employment pressure (7, 8). Therefore, it is crucial to explore the factors influencing SRH of medical students. Although the evidences for the association of SRH with lifestyle (e.g., physical activity, diet, sleep behavior) have been validated in the general population (9, 10), the evidence in the medical student was insufficient and required further study.

Sleep behavior is involved in the regulation of individual metabolism and energy balance, and is an important part of the biological rhythm mechanism. Previous studies have shown a strong association between sleep duration and SRH (11). However, most current studies were performed in the middle-aged and elderly population. Sleep behavior changes with age, and the health effects of the recommended sleep duration may be different for different age groups (12). There were few and inconsistent reports on how sleep duration affects health outcomes in young individuals who do not yet have chronic diseases. Some studies suggested a *U*-shaped association between sleep duration and suboptimal SRH (13), and others suggested that only short sleep is associated with suboptimal SRH (14). To the best of our knowledge, previous studies have not controlled for confounding effect of circadian rhythm-related factors such as chronotype, daytime napping, snacking after dinner. Previous studies have shown that poor sleep quality was another risk factor for SRH, and there was a linear association (15). But this also did not take into account the confounding factors of biological rhythms, so the results need further verification. In addition, the joint effects of sleep duration and sleep quality in young individuals have rarely been reported, especially in medical students.

In this study, from the perspective of circadian rhythm, we aimed to compare the differences in behavioral habits and SRH of medical students with different sleep duration; and explored the independent and joint effects of sleep duration and sleep quality on suboptimal SRH after controlling for confounding factors including circadian rhythm-related factors, to provide a scientific basis for the prevention of suboptimal SRH in medical students from sleep behavior perspectives.

## Methods

### Study design and participants

This cross-sectional study was performed in a medical university in eastern China, from April to September 2021. The questionnaire was designed by experts in the fields of epidemiology, medical statistics, and sociology according to other large-scale cohort studies (6, 16–19), including basic demographics, sleeping behaviors, other lifestyle factors, biorhythm variables, and health status. We used proportional stratified cluster random sampling method to recruit undergraduates of various majors. Firstly, we stratified students according to majors (clinical medicine, nursing and others), and then randomly selected several classes by grade in each major. The number of classes was determined according to the proportion of the number of students in the major to that of the whole university. All students in the selected classes were invited to participate in the survey. This study is part of a cross-sectional study, so the sample size is calculated by the following formula:


$$\begin{array}{l} N=\frac{u_{\alpha /2}^{2}\times P_{0}\left(1-P_{0}\right)}{\delta^{2}}\times deff\times stratification\; \end{array}$$

According to previous relevant studies (5, 6), the proportion of suboptimal SRH (*P*_0_) was 35.6–54.6%. Suppose δ = 10% × *P*_0_, α = 0.05, *u*_α/2_ = 1.96, design efficiency *deff* = 1.5, stratification = 3, non-response rate = 10%, the total sample size should be 1,067–2,317. We actually recruited 1,635 medical students (1,524 valid response), which was within the target sample size.

Inclusion criteria: 18 years of age and above, medical-related majors, and able to cooperate with the investigator (submission of questionnaires was regarded as informed consent). Exclusion criteria: those who could not participate in the survey due to various reasons, those with missing self-rated health or missing sleep duration. We excluded those who had missing information on sleep behaviors (*n* = 26), and those who had missing SRH information (*n* = 85), leaving 1,524 participants (mean age = 19.9 years, SD = 1.2 years; 59.1% female) in our main analysis.

In order to evaluate the test-retest reliability of measurement methods for sleep duration, sleep quality and self-rated health in the questionnaire, we randomly selected 163 medical students (95 were female) from the source population, and conducted a repeated survey in June 2022. To verify the calibration validity of sleep quality, we used the Pittsburgh Sleep Quality Index (PSQI) as the reference method. And to assess the validity of SRH, we collected 103 medical students in the source population and used the Self-Rated Health Measurement Scale (SRHMS) as the reference method, which includes 48 items and has a Cronbach's α coefficient of 0.93 (20). We also selected 60 medical students from the source population and asked them to record sleep diary for five consecutive days (including both weekdays and weekends, a total of 267 valid diaries were collected in 5 days) to evaluate the validity of sleep duration. The sample size needed for our validation study was shown in the Supplementary Figure 1.

This study was approved by the Ethics Committee of Wenzhou Medical University (ethics approval number: 2021-022). The online submission of the questionnaire by all participants was deemed informed consent. The entire investigation process was conducted in accordance with the principles of the Declaration of Helsinki.

### Assessment of sleep behaviors

**Figure 1:**
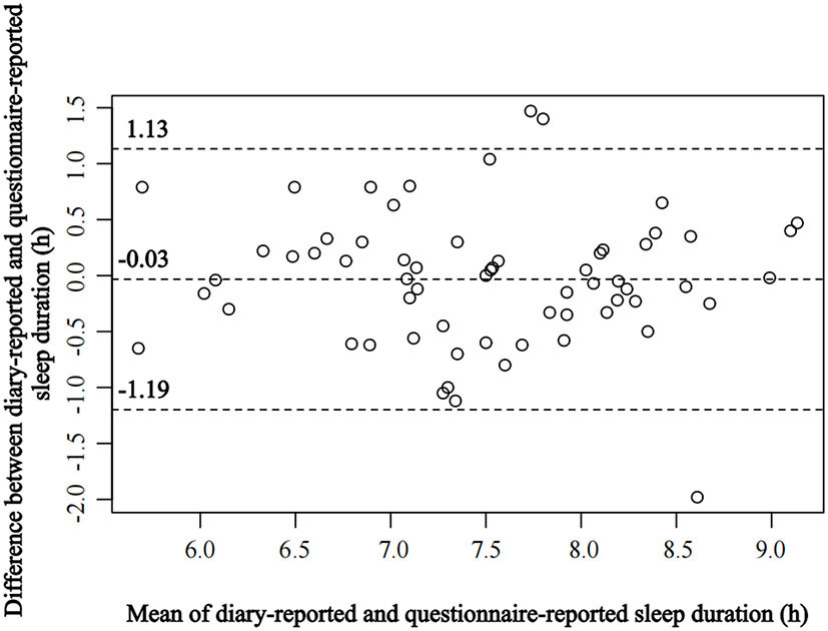
Bland-Altman plot of diary-reported and questionnaire-reported sleep durations.

The sleep duration was measured by the item: “How many hours do you usually sleep per night?”, and the options were divided into 7 groups: ≤ 5, 6, 7, 8, 9, 10, and ≥11 h (*n* = 28, 268, 784, 403, 34, 4, and 3). According to previous study (21), they were combined into 3 groups: ≤ 6 h of sleep duration (short sleep), 7 h of sleep duration, ≥8 h of sleep duration (long sleep). In secondary analyses, we divided sleep duration into 4 groups (≤ 6, 7, 8, ≥9 h). In our validation study, among 163 students, the mean ± SD of sleep duration at the first survey was 7.20 ± 0.87 h, and the mean sleep duration of the second survey was 7.49 ± 0.92 h, and there was no statistical difference between these two surveys (*P* > 0.05). The test-retest correlation of sleep duration between the first and the second surveys was 0.84. The mean ± SD of sleep duration in the sleep diary was 7.49 ± 0.86 h, and the correlation coefficient (*r*) between diary-reported and questionnaire-reported sleep duration was 0.78. We also evaluated the agreement between these two methods using Bland-Altman plot (Figure 1). This indicates that our questionnaire-reported sleep duration has good reliability and validity. Furthermore, the correlation between weekends and weekdays sleep duration in the questionnaire was 0.83, which was consistent with previous study (22), indicating that despite the observation of compensatory sleep on weekends, this sleep duration variability was negligible in this study. Previous studies also have found a good correlation between self-rated sleep duration and objective methods [e.g., wrist actigraphy, sleep diary; (23)], and can truly reflect the population's sleep duration.

The sleep quality was evaluated by the item: “How do you think your sleep quality is?”, and the options were divided into 5 groups: very good, good, fair, poor, and very poor (*n* = 289, 602, 520, 90, and 23). Further, they were combined into 3 groups (good, fair, and poor) in analysis. In order to verify the validity of the item for evaluating sleep quality, we used the Pittsburgh Sleep Quality Index Questionnaire (PSQI) as the reference method, and found that the correlation (*r*_*s*_) between the sleep quality obtained by the items in the questionnaire and the sleep quality obtained by the PSQI scale was 0.65. And 87.0% of the participants had consistent response on sleep quality in the first and second surveys, and the test-retest correlation of sleep quality between these two surveys was 0.47, indicating moderate reproducibility of questionnaire-reported sleep quality (24). Studies had shown that the single self-rated sleep quality item in the PSQI can distinguish between good and poor sleep quality (25).

### Assessment of self-rated health

SRH is a subjective measure of health status that integrates a person's biological, psychological, social, and functional aspects; and has been widely used in epidemiological studies (1, 5, 26). In this study, SRH was assessed by five-point Likert scale of self-rated health corresponding: “How do you feel about your health in general?” (27, 28), and the options were divided into 5 groups (1 = very good, 2 = good, 3 = fair, 4 = poor, 5 = very poor) (6). Referring to the standards of other international cohort study (29), those with a score of 3–5 were defined as suboptimal SRH, and those with a score of 1 or 2 were defined as good SRH. Psychometric performance of this assessment has been demonstrated in previous studies (30, 31). Although self-rated health was assessed with only a single item, the expertise and competence of medical students facilitated the acquisition of relatively reliable information for this study. We found that the test-retest correlation of SRH between the first and the second survey was 0.50, and 70.4% of the respondents had consistent responses in the two measurements, and the weighted kappa coefficient was 0.41, indicating moderate reproducibility of self-rated health. We also used the Self-Rated Health Measurement Scale (SRHMS) to assess the health status, and the Spearman correlation coefficient (*r*_*s*_) between SRH and SRHMS was 0.74. Moreover, we assessed the medical history during the past year by the question “Have you been to the hospital in the past year” in the original survey; and found that the proportion of suboptimal SRH with medical history in the past year was higher than that of medical students without medical history (50.2 vs. 36.1%). All these findings indicated that the method for evaluating SRH in this study had acceptable reliability and validity.

### Assessment of covariates

The demographic characteristics included sex, grade [sophomore and below, Junior year and above; (32)], parental education (elementary school or below, junior middle school, senior high school, university or above), major (clinical medicine, others), residential district (city, town, village), one-child family (yes, no). Lifestyle and biorhythm variables included physical activity (continuous), sedentary behavior (continuous), smoking (yes, no), drinking (yes, no), sleep latency (≤ 15, 16–29, ≥30 min), daytime napping (0, 1–30, >30 min), chronotype (morning type, neutral type, evening type), snacking after dinner (yes, no), meal time (ordinal), and maximum meal (breakfast, lunch or dinner).

Social-economic status was evaluated by parental education. The parental education refers to the educational level of the father or mother with higher educational level, and divided into 4 groups (elementary school or below, junior middle school, senior high school, university or above). Physical activity and sedentary behavior were obtained from part of the International Physical Activity Questionnaire-Short Form (IPAQ-SF) scale (33). Studies have shown that more than 2 h of physical activity and <9 h of sedentary behavior will reduce the incidence of disease (34). So physical activity was divided into 2 groups (<2, ≥2 h) in studies. Similarly, sedentary behavior was divided into 2 groups (<9, ≥9 h). Sleep latency was divided into three groups according to PSQI classification criteria (≤ 15, 16–29, ≥30 min) (35). Daytime napping was divided into three groups (0, 1–30, >30 min) (36). Chronotype was an indicator for assessing an individual's circadian rhythm status (37). The question “People can be divided into early risers and late sleepers, which type do you think you belong to?” was asked, and the options were divided into 3 groups (morning type, neutral type, and evening type) (38, 39). Studies have shown that the self-rated chronotype had a good correlation with the total score of the Morning and Evening Questionnaire-5 (MEQ-5) (*r* = 0.72) (40), which can better reflect the individual's circadian rhythm state. The time of three meals was divided into breakfast (<8:00, ≥8:00 AM), lunch (<12:00, ≥12:00), and dinner (<6:00, ≥6:00 PM) according to the medical university teaching schedule to which the participant belongs. Evidence has shown that people who eat too much at dinner would get less sleep (41), so we divided maximum meals into 2 groups (dinner or not). Height and weight were the most recent measurements reported by the participants, and body mass index (BMI) was calculated as BMI = weight (kg)/height (m)^2^. We used the WS/T 428-2013 (China) standard to divide BMI into 4 groups (36): underweight (BMI <18.5 kg/m^2^), normal (18.5 ≤ BMI <24 kg/m^2^), overweight (24 ≤ BMI <28 kg/m^2^) and obese (BMI ≥ 28 kg/m^2^). Evidence has shown that overweight and obesity were more harmful to health than underweight (42), so we divided body types into 2 groups: underweight or normal (BMI <24 kg/m^2^), overweight or obese (BMI ≥ 24 kg/m^2^).

### Statistical analyses

Continuous data among students with different sleep durations were compared by ANOVA if normally distributed or the Kruskal–Wallis test otherwise. The Chi-square test was used for nominal data and the Kruskal–Wallis test was used for ordinal data. For the correlation between two quantitative variables, we used the Pearson correlation or Spearman correlation as appropriate; and the consistency between the two categorical variables was evaluated by the weighted kappa coefficient. We also evaluated the agreement of sleep duration obtained from sleep diary or from questionnaire using Bland-Altman analysis. We used a restricted cubic spline to explore the form of the relationship between sleep duration and suboptimal SRH. Logistic regression was used to analyze the independent and joint effects of sleep duration and sleep quality on suboptimal SRH. Results were expressed as odds ratio (*OR*) and 95% confidence interval (*CI*). The model I adjusted for demographic and lifestyle characteristics including sex, grade, major, residential district, parental education, physical activity, and sedentary behavior. To further control the potential confounding of biorhythmic variables, we adjusted for dinner time, maximum meal, chronotype, and daytime napping in Model II.

To explore the consistency of the association of sleep duration, sleep quality with suboptimal SRH in medical students, we conducted stratified analyses according to sex, major, grade, sleep latency, daytime napping, sedentary behavior, physical activity, chronotype, and maximum meal. The *P* for trend was obtained by assigning the ordinal value to each sleep duration and sleep quality categories and modeling them as continuous variables. The interactions between sleep quality, sleep quality and stratification factors were assessed by likelihood ratio tests comparing the models with and without the multiplicative interaction terms. Then, we investigated the joint association of sleep duration and sleep quality on suboptimal SRH. In this multivariable-adjusted logistic model, we combined the 3 groups of sleep quality with the 3 groups of sleep duration to form 9 subgroups, with sleep duration ≥8 h/night and good sleep quality as reference.

In sensitivity analyses, we restricted participants within medical students who did not smoke, drink alcohol, snack after dinner, or those who had dinner ≤ 6:00 PM, bedtime ≥11:00 PM, waketime ≥7:00 AM, or those who were underweight/normal. In addition, we performed multiple imputations for covariates with missing values to test the robustness of our results. All statistical analyses were implemented using SAS software, version 9.4 and R (http://www.R-project.org). A 2-sided *P-*value <0.05 was considered statistically significant.

## Results

The mean age of the 1,524 participants was 19.6 years (SD = 1.2 years; 59.1% female). Of them, 652 participants (42.8%) had suboptimal SRH, with females reporting higher rates of suboptimal SRH than males (45.6% for females vs. 38.8% for males, χ^2^ = 6.9, *P* = 0.009). From the distribution of sleep duration per night, most medical students slept 7 h (*n* = 84, 51.5%), 19.4% of them (*n* = 296) had short sleep (≤ 6 h), and 29.1% of them (*n* = 444) had long slept (≥8 h).

### Basic characteristics of medical students according to sleep duration

Compared with medical students who slept ≥8 h/night, those who slept ≤ 6 h/night had a higher proportion of junior year and above (159 [35.8%] for those with sleep ≥8 h vs. 172 [58.1%] for those with sleep ≤ 6 h), had a higher proportion of evening types (171 [38.5%] for those with sleep ≥8 h vs. 141 [47.6%] for those with sleep ≤ 6 h), had a higher proportion of sleeping after 11:00 PM (277 [62.4%] for those with sleep ≥8 h vs. 261 [88.2%] for those with sleep ≤ 6 h), a higher proportion of poor sleep quality (17 [3.8%] for those with sleep ≥8 h vs. 47 [15.9%] for those with sleep ≤ 6 h), a higher proportion of long sleep latency (89 [22.3%] for those with sleep ≥8 h vs. 98 [36.4%] for those with sleep ≤ 6 h), a higher proportion of dinner time ≥6:00 PM (69 [15.5%] for those with sleep ≥8 h vs. 78 [26.4%] for those with sleep ≤ 6 h) and a higher proportion of maximum meal was dinner (78 [17.6%] for those with sleep ≥8 h vs. 77 [26.0%] for those with sleep ≤ 6 h; Table 1).

**Table 1:** Basic characteristics and biorhythmic factors of medical students by sleep duration (*n* = 1524).

**Characteristics**	**Sleep duration, h/night**			***χ^2^*/*H***	***P-*value**
	**≤ 6 (*n* = 296)**	**7 (*n* = 784)**	**≥8 (*n* = 444)**		
Female, *n* (%)	186 (62.8)	464 (59.2)	250 (56.3)	3.14	0.208
Junior year and above, *n* (%)	172 (58.1)	343 (43.8)	159 (35.8)	35.9	**<0.001**
One-child family, *n* (%)	151 (51.0)	359 (45.8)	199 (44.8)	3.09	0.214
Clinical medicine major, *n* (%)	142 (48.0)	400 (51.0)	221 (49.8)	0.82	0.664
Parental education level, *n* (%)				2.26	0.323
Elementary school or below	35 (11.8)	65 (8.3)	28 (6.3)		
Junior middle school	105 (35.5)	299 (38.1)	159 (35.8)		
Senior high school	92 (31.1)	221 (28.2)	160 (36.0)		
University or above	64 (21.6)	199 (25.4)	97 (21.9)		
Residential district, *n* (%)				2.50	0.645
City	123 (41.6)	290 (37.1)	169 (38.1)		
Town	55 (18.6)	171 (21.9)	98 (22.0)		
Village	118 (39.8)	320 (41.0)	177 (39.9)		
Underweight/normal, *n* (%)	241 (85.2)	660 (88.1)	376 (88.5)	2.04	0.361
Chronotype, *n* (%)				9.90	**0.007**
Morning type	68 (23.0)	208 (26.5)	144 (32.4)		
Neutral type	87 (29.4)	226 (28.8)	129 (29.1)		
Evening type	141 (47.6)	350 (44.7)	171 (38.5)		
Bedtime ≥ 11:00 PM, *n* (%)	261 (88.2)	623 (79.5)	277 (62.4)	74.68	**<0.001**
Waketime ≥ 7:00 AM, *n* (%)	169 (57.3)	576 (73.5)	361 (81.3)	52.01	**<0.001**
Sleep quality, *n* (%)				82.03	**<0.001**
Good	120 (40.5)	451 (57.5)	320 (72.1)		
Fair	129 (43.6)	284 (36.2)	107 (24.1)		
Poor	47 (15.9)	49 (6.3)	17 (3.8)		
Sleep latency, min, *n* (%)				27.01	**<0.001**
≤ 15	125 (46.5)	391 (53.9)	259 (64.9)		
16–29	46 (17.1)	133 (18.3)	51 (12.8)		
≥30	98 (36.4)	202 (27.8)	89 (22.3)		
Daytime napping, min, *n* (%)				7.46	0.114
0	73 (25.2)	178 (23.5)	106 (24.5)		
1–30	116 (40.0)	325 (42.8)	153 (35.3)		
>30	101 (34.8)	256 (33.7)	174 (40.2)		
Screen time, h, Median (*P_25_, P_75_*)	4.00 (2.50, 6.00)	4.00 (3.00, 6.00)	4.00 (2.50, 5.00)	4.49	0.201
Sedentary behavior ≥9 h, *n* (%)	114 (39.5)	281 (63.4)	133 (30.2)	7.51	**0.023**
Physical activity <2 h, *n* (%)	196 (68.8)	569 (75.5)	307 (71.7)	5.26	0.072
Breakfast time <8:00 AM, *n* (%)	226 (76.4)	550 (70.2)	248(55.9)	45.09	**<0.001**
Lunch time ≥12:00 AM, *n* (%)	67 (22.6)	151 (19.3)	105 (23.7)	3.73	0.155
Dinner time ≥6:00 PM, *n* (%)	78 (26.4)	151 (19.3)	69 (15.5)	13.46	**0.001**
Snacking after dinner, *n* (%)	57 (19.3)	124 (15.8)	71 (16.0)	1.98	0.372
Maximum meal = dinner, *n* (%)	77 (26.0)	174 (22.2)	78 (17.6)	7.83	**0.020**

The Chi-square test was used for unordered categorical data and the Kruskal-Wallis test was used for ordinal data, and bold values indicated statistical significance *P* < 0.05.

### Independent effects of sleep duration and sleep quality on suboptimal self-rated health

**Figure 2:**
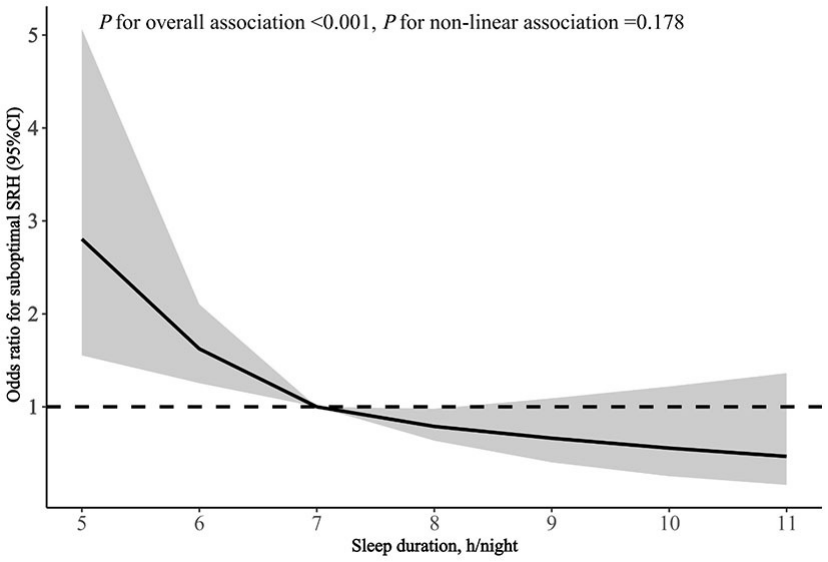
Spline curve for the association of sleep duration with suboptimal SRH among medical students. Adjusted for sex, grade (sophomore and below, Junior year and above), major (clinical medicine, others), parental education level (elementary school or below, junior middle school, senior high school, university or above), residential district (city, town, village), body type (underweight/normal, overweight/obese), sedentary behavior (<9, ≥9 h), physical activity (<2, ≥2 h), snacking after dinner (yes, no), chronotype (morning type, neutral type, evening type), daytime napping (0, 1–30, >30 min), sleep latency (≤ 15, 16–29, ≥30 min), dinner time (<6:00, ≥6:00 PM) and maximum meal (dinner, other).

Our study showed that the proportion of suboptimal SRH reported with sleep duration ≥8 h/night was the lowest (31.8% for those who sleep ≥8 h vs. 43.2% for those who sleep 7 h vs. 58.1% for those who sleep ≤ 6 h; χ^2^ = 50.5, *P* < 0.001; Table 2). Similarly, the proportion of suboptimal SRH reported with good sleep quality was the lowest (27.7% for those who have good sleep quality vs. 60.4% for those who have fair sleep quality vs. 80.5% for those who have poor sleep quality; χ^2^ = 214.2, *P* < 0.001; Table 2). In addition, the restricted cubic spline also showed that short sleep duration was associated with suboptimal SRH (*P* for overall <0.001, *P* for non-linear = 0.178; Figure 2).

**Table 2:** Association of sleep duration and sleep quality with suboptimal SRH among medical students.

**Sleep behaviors**	**Suboptimal SRH, *n* (%)**	^**a**^***OR*** **(95%*****CI*****)**		
		**Crude model**	**Adjusted model I**	**Adjusted model II**
Sleep duration, h
≥8	141 (31.8)	1.00	1.00	1.00
7	339 (43.2)	1.65 (1.29, 2.10)	1.60 (1.25, 2.05)	1.37 (1.03, 1.81)
≤ 6	172 (58.1)	2.99 (2.20, 4.06)	2.70 (1.97, 3.69)	2.24 (1.57, 3.19)
*P* for trend		<0.001	<0.001	<0.001
Sleep quality
Good	247 (27.7)	1.00	1.00	1.00
Fair	314 (60.4)	3.97 (3.16, 5.00)	4.04 (3.19, 5.11)	4.14 (3.13, 5.47)
Poor	91 (80.5)	10.79 (6.62, 17.57)	11.70 (7.10, 19.27)	11.50 (6.52, 20.29)
*P* for trend		<0.001	<0.001	<0.001

*OR*, odds ratio; *CI*, confidence interval.

^*a*^Model I was adjusted for sex, grade (sophomore and below, Junior year and above), major (clinical medicine, others), parental education level (elementary school or below, junior middle school, senior high school, university or above), residential district (city, town, village). Model II was additionally adjusted for chronotype (morning type, neutral type, evening type), daytime napping (0, 1–30, >30 min), sleep latency (≤ 15, 16–29, ≥30 min), dinner time (<6:00, ≥6:00 PM), snacking after dinner (yes, no), body type (underweight/normal, overweight/obese), sedentary behavior (<9 h, ≥9 h), and physical activity (<2 h, ≥2 h), maximum meal (dinner, other).

Compared with students sleeping for 8 h/night, the multivariable-adjusted *ORs* in the model I were 2.70 (95%*CI*: 1.97–3.69) for ≤ 6 h, 1.60 (95%*CI*: 1.25–2.05) for 7 h, respectively, *P* < 0.001 for trend. After further adjustment for biorhythmic factors such as chronotype, daytime napping, dinner time, and maximum meal (model II), the adjusted *ORs* were 2.24 (95%*CI*: 1.57–3.19) for those sleeping ≤ 6 h, 1.37 (95%*CI*: 1.03–1.81) for those sleeping 7 h, respectively (Table 2). For comparability with other studies, we used 7 h as the reference, and recalculated odds ratio (*ORs*) by multivariable-adjusted logistic regression. In model II, the *ORs* (95%*CI*) for short sleep (≤ 6 h), sleep 8 h, and long sleep (≥9 h) were 1.72 (1.26–2.35), 0.69 (0.51–0.92), and 1.00 (0.49–2.04), respectively, *P* < 0.001 for trend (Supplementary Table 1). We also explored the association between sleep quality and suboptimal SRH. Compared with medical students with good sleep quality, the students with fair or poor sleep quality had higher odds of suboptimal SRH, the multivariable-adjusted *ORs* in model I were 4.04 (95%*CI*: 3.19–5.11) for fair, 11.70 (95%*CI*: 7.10–19.27) for poor, respectively, *P* < 0.001 for trend. After further adjustment for the confounders in model II, the adjusted *ORs* were 4.14 (95%*CI*: 3.13–5.47) for those with fair sleep quality, 11.50 (95%*CI*: 6.52–20.29) for those with poor sleep quality, respectively (Table 2). The stratified analysis according to selected variables found consistent trends in the association between short sleep duration, sleep quality, and suboptimal SRH (Table 3). To further exclude the confounding of lifestyle factors, we performed sensitivity analyses. When we restricted participants to non-smokers, non-drinkers, or participants who do not snack after dinner, had dinner ≤ 6:00 PM, bedtime ≥11:00 PM, waketime ≥7:00 AM or those with underweight or normal weight, the association between sleep duration and suboptimal SRH was consistent (Table 4). Then to evaluate the effect of missing values on our results, we performed multiple imputations. In our study, the *n* (%) of missing value for sleep latency, body type, physical activity, daytime napping, sedentary behavior, residential district, and dinner time were 130 (8.5), 67 (4.4), 57 (3.7), 42 (2.8), 22 (1.4), 3 (0.2), and 2 (0.1), respectively. In multiple imputation, we included the above variables and also SRH, sleep duration, sleep quality, sex, grade, chronotype, snacking after dinner, and found that the association of sleep duration and sleep quality with suboptimal SRH remained unchanged (Supplementary Table 2).

**Table 3:** Association of suboptimal SRH with sleep duration and sleep quality, stratification analyses.

**Subgroup**	***n***	^**a**^***OR*** **(95%*****CI*****)**							
		**Sleep duration**				**Sleep quality**			
		**≤ 6 h**	**7 h**	**≥8 h**	***P* for interaction**	**Poor**	**Fair**	**Good**	***P* for interaction**
Sex					0.749				0.141
Female	900	2.57 (1.60, 4.12)	1.40 (0.96, 2.03)	1.00		18.08 (7.15, 45.73)	3.61 (2.52, 5.18)	1.00	
Male	624	1.78 (1.00, 3.15)	1.33 (0.85, 2.07)	1.00		9.18 (4.18, 20.15)	5.07 (3.20, 8.03)	1.00	
Major					0.876				0.425
clinical medicine	763	1.98 (1.18, 3.32)	1.19 (0.79, 1.79)	1.00		8.15 (3.54, 18.74)	4.59 (3.06, 6.89)	1.00	
others	761	2.45 (1.48, 4.04)	1.48 (0.99, 2.20)	1.00		14.09 (6.34, 31.29)	3.89 (2.61, 5.79)	1.00	
Grade					0.487				0.337
Freshman/sophomore	850	2.90 (1.74, 4.81)	1.59 (1.09, 2.32)	1.00		15.59 (7.03, 34.59)	4.85 (3.32, 7.08)	1.00	
Junior year and above	674	1.68 (1.00, 2.85)	1.08 (0.69, 1.68)	1.00		8.35 (3.65, 19.12)	3.78 (2.45, 5.85)	1.00	
Daytime napping, min					0.927				0.882
0	357	2.12 (1.01, 4.44)	1.67 (0.90, 3.09)	1.00		11.01 (3.53, 34.33)	3.85 (2.12, 7.01)	1.00	
1–30	594	2.72 (1.50, 4.93)	1.53 (0.96, 2.43)	1.00		14.74 (5.41, 40.15)	3.95 (2.52, 6.21)	1.00	
>30	531	2.04 (1.12, 3.73)	1.17 (0.73, 1.87)	1.00		10.30 (4.01, 26.46)	5.23 (3.20, 8.55)	1.00	
Sleep latency, min					0.175				0.063
≤ 15	775	2.18 (1.34, 3.55)	1.27 (0.87, 1.83)	1.00		14.29 (4.92, 41.50)	5.66 (3.76, 8.51)	1.00	
16–29	230	0.75 (0.28, 2.00)	0.71 (0.33, 1.53)	1.00		22.37 (2.48, 201.46)	1.75 (0.93, 3.30)	1.00	
≥30	389	3.65 (1.81, 7.35)	2.12 (1.18, 3.81)	1.00		9.82 (4.47, 21.57)	4.71 (2.73, 8.15)	1.00	
Sedentary behavior, h					0.148				0.450
< 9	974	1.63 (1.04, 2.56)	1.26 (0.90, 1.77)	1.00		14.28 (7.00, 29.13)	4.66 (3.28, 6.62)	1.00	
≥9	528	3.85 (2.04, 7.27)	1.71 (1.01, 2.91)	1.00		8.77 (3.28, 23.45)	3.67 (2.24, 6.02)	1.00	
Physical activity, h					0.333				0.052
<2	1072	2.36 (1.55, 3.59)	1.26 (0.91, 1.74)	1.00		7.90 (4.26, 14.68)	3.60 (2.62, 4.95)	1.00	
≥2	395	1.70 (0.83, 3.46)	1.73 (0.96, 3.13)	1.00		83.07 (15.16, 455.25)	7.45 (3.96, 14.02)	1.00	
Chronotype					0.611				0.067
Morning type	420	3.03 (1.41, 6.53)	1.73 (0.94, 3.18)	1.00		80.49 (8.94, 724.52)	6.61 (3.48, 12.53)	1.00	
Neutral type	442	2.11 (1.09, 4.07)	1.22 (0.72, 2.06)	1.00		11.95 (4.64, 30.76)	5.49 (3.18, 9.46)	1.00	
Evening type	662	2.07 (1.21, 3.54)	1.36 (0.89, 2.08)	1.00		7.23 (3.30, 15.86)	2.93 (1.96, 4.39)	1.00	
Maximum meal					0.256				0.520
Dinner	329	3.34 (1.50, 7.42)	2.08 (1.06, 4.09)	1.00		7.07 (2.15, 23.21)	3.54 (1.91, 6.55)	1.00	
Other	1195	2.02 (1.34, 3.03)	1.18 (0.86, 1.61)	1.00		13.38 (6.93, 25.84)	4.62 (3.34, 6.37)	1.00	

*OR*, odds ratio; *CI*, confidence interval.

^*a*^Adjusted for sex, grade (sophomore and below, Junior year and above), major (clinical medicine, others), parental education (elementary school or below, junior middle school, senior high school, university or above), residential district (city, town, village), snacking after dinner (yes, no), body type (underweight/normal, overweight/obese), sedentary behavior (<9, ≥9 h), physical activity (<2, ≥2 h), chronotype (morning types, neutral types, evening types), daytime napping (0, 1–30, >30 min), sleep latency (≤ 15, 16–29, ≥30 min), dinner time (<6:00, ≥6:00 PM), and maximum meal (dinner, other), except for the stratification factor itself.

**Table 4:** Sensitivity analyses of the association of suboptimal SRH with sleep duration and sleep quality.

**Limiting population to:**	***n***	^**a**^***OR*** **(95%*****CI*****)**					
		**Sleep duration**			**Sleep quality**		
		**≤ 6 h**	**7 h**	**≥8 h**	**Poor**	**Fair**	**Good**
Non-smokers	1497	2.26 (1.58, 3.24)	1.33 (1.00, 1.77)	1.00	12.09 (6.78, 21.58)	4.21 (3.18, 5.58)	1.00
Non-drinkers	1474	2.33 (1.62, 3.36)	1.45 (1.09, 1.93)	1.00	12.52 (6.91, 22.69)	4.24 (3.19, 5.63)	1.00
No snack after dinner	1272	2.30 (1.55, 3.40)	1.32 (0.97, 1.79)	1.00	11.01 (5.99, 20.23)	4.20 (3.09, 5.71)	1.00
Dinner time ≤ 6:00 PM	1224	2.26 (1.51, 3.37)	1.37 (1.00, 1.87)	1.00	12.51 (6.54, 23.91)	4.19 (3.06, 5.74)	1.00
Underweight/normal	1277	2.47 (1.68, 3.63)	1.50 (1.11, 2.03)	1.00	13.20 (6.96, 25.05)	4.05 (3.00, 5.45)	1.00
Bedtime ≥11:00 PM	1161	2.13 (1.42, 3.19)	1.26 (0.90, 1.76)	1.00	10.29 (5.54, 19.10)	4.16 (3.04, 5.68)	1.00
Waketime ≥7:00 AM	1106	2.30 (1.50, 3.54)	1.51 (1.11, 2.05)	1.00	10.15 (5.35, 19.25)	4.89 (3.55, 6.75)	1.00

*OR*, odds ratio; *CI*, confidence interval; BMI, body mass index.

^*a*^Adjusted for sex, grade (sophomore and below, Junior year and above), major (clinical medicine, others), parental education level (elementary school or below, junior middle school, senior high school, university or above), residential district (city, town, village), snacking after dinner (yes, no), body type (underweight/normal, overweight/obese), sedentary behavior (<9, ≥9 h), physical activity (<2, ≥2 h), chronotype (morning type, neutral type, evening type), daytime napping (0, 1–30, >30 min), sleep latency (≤ 15, 16–29, ≥30 min), dinner time (<6:00, ≥6:00 PM) and maximum meal (dinner, other).

### Joint effects of sleep duration and sleep quality on suboptimal self-rated health

In addition, we explored the joint effects of sleep quality and sleep duration on suboptimal SRH. Taking participants who reported sleeping ≥8 h/night and good sleep quality as a reference, the multivariable-adjusted *ORs* of other groups were all >1, of which the group with sleep ≤ 6 h and poor sleep quality had the highest odds of suboptimal SRH (*OR* 23.12, 95%*CI*: 8.33–64.17; Table 5).

**Table 5:** Joint effects of sleep duration and sleep quality on suboptimal self-rated health.

**Sleep quality**	**Sleep duration, h/night**	**Suboptimal SRH, *n* (%)**	**^a^*OR* (95%*CI*)**	***P* for interaction**
Good	≥8	68 (21.3)	1.00	0.297
	7	138 (30.6)	1.43 (0.97, 2.09)	
	≤ 6	41 (34.2)	1.49 (0.87, 2.56)	
Fair	≥8	61 (57.0)	5.59 (3.29, 9.50)	
	7	162 (57.0)	4.52 (2.97, 6.89)	
	≤ 6	91 (70.5)	7.23 (4.23, 12.33)	
Poor	≥8	12 (70.6)	6.74 (1.98, 22.93)	
	7	39 (76.6)	14.28 (6.20, 32.85)	
	≤ 6	40 (85.1)	23.12 (8.33, 64.17)	

^*a*^Adjusted for sex, grade (sophomore and below, Junior year and above), major (clinical medicine, others), parental education level (elementary school or below, junior middle school, senior high school, university or above), residential district (city, town, village), body type (underweight/normal, overweight/obese), sedentary behavior (<9, ≥9 h), physical activity (<2, ≥2 h), snacking after dinner (yes, no), chronotype (morning type, neutral type, evening type), daytime napping (0, 1–30, >30 min), sleep latency (≤ 15, 16–29, ≥30 min), dinner time (<6:00, ≥6:00 PM) and maximum meal (dinner, other).

## Discussion

In this cross-sectional study of medical students, we observed that short sleep duration (≤ 6 h) was significantly associated with suboptimal SRH, but not long sleep. We also observed that sleep quality was highly correlated with suboptimal SRH in a dose-response relationship. In addition, we found that sleep duration and sleep quality had significant joint effects on suboptimal SRH. All these associations were independent of biorhythmic variables, such as chronotype, dinner time, and maximum meal, which most current studies have not considered.

This study found that 42.8% of medical students reported suboptimal SRH, which was higher than the level of suboptimal SRH reported in the general population (43, 44). Additionally, the difference in the reporting rate of suboptimal SRH among young adults between sex was controversial. The European Health Behavior Survey (6) showed no difference in the reporting rate of SRH between the sexes. But we found that female medical students reported a higher rate of suboptimal SRH than males, which is consistent with findings from the European Health Interview Survey (1). One of the reasons for the difference may be related to the heavy academic and learning pressure of medical students, and the other reason is related to the different definitions of suboptimal SRH in different studies. We found that 296 (accounting for 19.4%) of medical students suffered from short sleep, which was lower than other college students (21–46%) (6, 39, 45). This may be related to the rules and regulations of the university to which the interviewee belongs. As far as we know, the library of this university is closed at 10:30 PM, the study room is turned off at 11:00 PM at night, and the dormitory is set up with access control at 11:00 PM. Furthermore, our study found that average daily screen time of medical students was ~4 h, which was lower than the level of previous studies (7 h) (46). This may be another reason that medical students have a lower rate of short sleep than other university students. Therefore, even though medical students face pressures such as clinical practice and further studies, their short sleep rate is lower than other studies.

Unlike the *U*-shaped association between sleep duration and suboptimal SRH in general adults (5, 16, 43), we found that short sleep duration was significantly associated with suboptimal SRH, but not long sleep. And this association remained robust after adjusting for demographic characteristics and biorhythms. The reason for the difference may be related to the large variability of sleep duration in different previous studies. In our study, most participants slept 7–8 h per night (77.9%). But in previous studies, the proportion of sleep 7–8 h was between 42.9 and 62.1% (16, 43, 47), and the proportion of participants with short or long sleep duration was very high. Few studies among undergraduates (6, 45) and adolescents (48) also proved that short sleep (≤ 6 h) was significantly associated with suboptimal SRH, while long sleep (≥9 h) was non-significant. It may be related to the confounding effects of biological rhythm factors such as maximum meal, and chronotype that were not controlled in previous studies. In this study, we found that after controlling for these factors, short sleep (≤ 6 h) was still associated with suboptimal SRH, while long sleep (≥9 h) was not statistically significant. The result was consistent with the joint statement of the American Academy of Sleep Medicine and the Sleep Research Society, indicating optimal sleep duration standard for young people might be different from that of middle-aged and elderly people and that regular long sleep duration is suitable for them (49).

The previous study had shown that sleep quality was an important predictor of SRH in adults (50). Our study also found that sleep quality had a dose-response association with suboptimal SRH, the better the sleep quality, the lower the proportion of suboptimal SRH. This was consistent with previous findings that sleep quality was linearly associated with SRH (15). These data indicated that the excellent sleep quality of young medical students or young individuals played an important role in health.

Moreover, it is worth noting that joint effects of sleep duration and sleep quality were significantly associated with suboptimal SRH, which was consistent with another cohort study (36). However, an observational study showed poor sleep quality was associated with suboptimal SRH in long sleep duration but not short sleep duration (*n* = 1,304, 18–79 years) (15). In our study, this association might exist because we used a more homogeneous sample (18–26 years) while adjusting for biorhythm covariates in the analysis.

Existing evidence suggests that short sleep duration and poor sleep quality can lead to increased fatigue (51, 52), adverse effects on endocrine function, the immune system (53), blood sugar regulation (54), and cognitive function (55). Of course, suboptimal health might also lead to short or long sleep or poor sleep quality. But this was unlikely in this study because medical students are a younger adult population with a lower probability of having health problems. These data indicate that medical students or young adults should ensure adequate sleep duration and good sleep quality for optimal health.

There are several limitations to our study. Although we evaluated the relationship between sleep duration and suboptimal SRH under the condition of comprehensive control of various confounding factors, and we selected a relatively young adult population, medical students as the research participants, limiting the problems of reverse causality that may exist in the middle-aged and elderly population in previous studies due to changes in sleep behavior caused by chronic diseases. The association between sleep duration and suboptimal SRH could not be interpreted as a causal relationship because the data were cross-sectional and our results only suggest that poor sleep health is a marker or correlate of suboptimal SRH. In addition, sleep duration, sleep quality, and other variables were collected through questionnaires. Although we designed the SRH questionnaire with reference to many large cohort studies (6, 18, 36), and conducted detailed reliability and validity assessments on variables such as sleep duration, sleep quality, and self-rated health, the variable acquisition method was relatively subjective, and the information was not as accurate as the objective evaluation method. We also did not monitor other residual confounding such as sleep duration variability, social jetleg, the number of wake-ups from sleep, fatigue and stress. Therefore, further cohort study among medical students is needed to confirm the relationship between sleep behavior and health.

In conclusion, based on the perspective of circadian rhythm, this study further verified the association between sleep quantity, quality and health in medical students, and found the rate of suboptimal SRH in medical students was higher than that in the general population, short sleep and poor sleep quality were independently and jointly associated with higher odds of suboptimal SRH, which was consistent with previous studies among college students and young adults, but was different from those in middle-aged and older populations. Therefore, education on sleep hygiene among medical students should be strengthened, and adequate and high-quality sleep should be advocated to prevent adverse health events. Furthermore, governments and universities should pay close attention to the sleep behaviors of medical students and young adults, conduct better cohort studies on sleep behaviors, and formulate recommendations for healthy sleep behaviors to promote health.

## Data availability statement

The raw data supporting the conclusions of this article will be made available by the authors, without undue reservation.

## Ethics statement

The studies involving human participants were reviewed and approved by Ethics Committee of Wenzhou Medical University, Wenzhou Medical University. The patients/participants provided their written informed consent to participate in this study.

## Author contributions

PD and HS designed the study and analyzed the data. JL, XY, and HS collected the data. PD drafted the manuscript. HC, XY, and CZ supervised the study. All authors contributed to revising the manuscript and approved the submitted version.

## Funding

This study was supported by grants from the National Social Science Foundation of China (21BRK021), Key Project of Philosophy and Social Science Planning Project in Zhejiang Province, China (21NDJC013Z), National College Students Innovation and Entrepreneurship Training Program, China (202010343008), and University Students Science and Technology Innovation Activity Plan in Zhejiang Province (2022R413C079).

## Conflict of interest

The authors declare that the research was conducted in the absence of any commercial or financial relationships that could be construed as a potential conflict of interest.

## Publisher's note

All claims expressed in this article are solely those of the authors and do not necessarily represent those of their affiliated organizations, or those of the publisher, the editors and the reviewers. Any product that may be evaluated in this article, or claim that may be made by its manufacturer, is not guaranteed or endorsed by the publisher.
