# Supplementary material for: Independent and joint effects of sleep duration and sleep quality on suboptimal self-rated health in medical students: A cross-sectional study
Source: Front Public Health. 2022 Oct 6;10:957409. doi: 10.3389/fpubh.2022.957409 (PMC9583520; doi:10.3389/fpubh.2022.957409)

**Supplemental Table 1 Association of sleep duration with suboptimal SRH among medical students**

| Sleep duration,  h/night | Suboptimal SRH, *n* (%) | *OR* (95%*CI*) | | |
| --- | --- | --- | --- | --- |
|  |  | Crude | Model I | Model II |
| ≤6 | 172 (58.1) | 1.82 (1.38, 2.38) | 1.79 (1.36, 2.36) | 1.72 (1.26, 2.35) |
| 7 | 339 (43.2) | 1.00 | 1.00 | 1.00 |
| 8 | 125 (31.0) | 0.59 (0.45, 0.76) | 0.59 (0.46, 0.76) | 0.69 (0.51, 0.92) |
| ≥9 | 16 (39.0) | 0.84 (0.44, 1.59) | 0.81 (0.42, 1.54) | 1.00 (0.49, 2.04) |
| *P* for trend |  | <0.001 | <0.001 | <0.001 |

*OR*, odds ratio; *CI*, confidence interval.

Model I was adjusted for sex, grade (sophomore and below, Junior year and above), major (clinical medicine, others), parental education level (elementary school or below, junior middle school, senior high school, university or above), residential district (city, town, village). Model II was additionally adjusted for chronotype (morning types, neutral types, evening types), daytime napping (0, 1-30, >30 min), sleep latency (≤15, 16-29, ≥30 min), dinner time (<6:00, ≥6:00PM), snacking after dinner (yes, no), body type (underweight/normal, overweight/obese), sedentary behavior (<9h, ≥9h), and physical activity (<2h, ≥2h), maximum meal (dinner, other).

**Supplemental Table 2 Associations of sleep duration, sleep quality with suboptimal self-rated health after imputation**

| Sleep behaviors | Suboptimal SRH, *n* (%) | ^a^ *OR* (95%*CI*) | *P* for trend |
| --- | --- | --- | --- |
| Sleep duration, h |  |  | <0.001 |
| ≥8 | 141 (31.8) | 1.00 |  |
| 7 | 339 (43.2) | 1.44 (1.11, 1.85) |  |
| ≤6 | 172 (58.1) | 2.30 (1.66, 3.18) |  |
| Sleep quality |  |  | <0.001 |
| Good | 247 (27.7) | 1.00 |  |
| Fair | 314 (60.4) | 3.81 (2.97, 4.91) |  |
| Poor | 91 (80.5) | 10.90 (6.48, 18.33) |  |

^a^ Adjusted for sex, grade (sophomore and below, Junior year and above), major (clinical medicine, others), parental education (elementary school or below, junior middle school, senior high school, university or above), residential district (city, town, village), snacking after dinner (yes, no), body type (underweight/normal, overweight/obese), sedentary behavior (< 9h, ≥ 9h), physical activity (< 2h, ≥ 2h), chronotype (morning types, neutral types, evening types), daytime napping (0, 1-30, >30 min), sleep latency (≤15, 16-29, ≥30 min), dinner time (< 6:00, ≥ 6:00PM), snacking after dinner (yes, no) and maximum meal (dinner, other).

**Supplemental Figure 1 Sample size estimation for correlation studies**


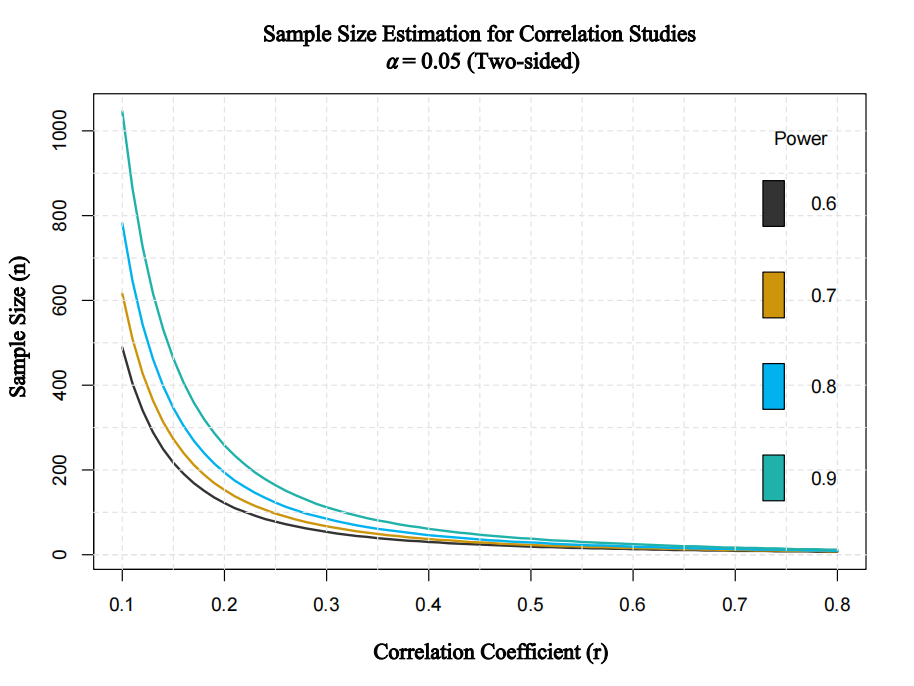

Supplement: Supplementary file 1 [file Data_Sheet_1.docx]
